# A resilient type-III broken gap Ga_2_O_3_/SiC van der Waals heterogeneous bilayer with band-to-band tunneling effect and tunable electronic property

**DOI:** 10.1038/s41598-024-63354-8

**Published:** 2024-06-03

**Authors:** Naim Ferdous, Md. Sherajul Islam, Jeongwon Park

**Affiliations:** 1 Department of Electrical and Biomedical Engineering, University of Nevada, Reno, NV 89557 USA; 2 Department of Electrical and Electronic Engineering, Khulna University of Engineering and Technology, Khulna, 9203 Bangladesh; 3 School of Electrical Engineering and Computer Science, University of Ottawa, Ottawa, ON K1N6N5 Canada

**Keywords:** Nanoscale devices, Nanoscale materials, Chemistry, Physics

## Abstract

The potential of van der Waals (vdW) heterostructure to incorporate the outstanding features of stacked materials to meet a variety of application requirements has drawn considerable attention. Due to the unique quantum tunneling mechanisms, a type-III broken-gap obtained from vdW heterostructure is a promising design strategy for tunneling field-effect transistors. Herein, a unique Ga_2_O_3_/SiC vdW bilayer heterostructure with inherent type-III broken gap band alignment has been revealed through first-principles calculation. The underlying physical mechanism to form the broken gap band alignment is thoroughly studied. Due to the overlapping band structures, a tunneling window of 0.609 eV has been created, which enables the charges to tunnel from the VBM of the SiC layer to the CBM of the Ga_2_O_3_ layer and fulfills the required condition for band-to-band tunneling. External electric field and strain can be applied to tailor the electronic behavior of the bilayer heterostructure. Positive external electric field and compressive vertical strain enlarge the tunneling window and enhance the band-to-band tunneling (BTBT) scheme while negative electric field and tensile vertical strain shorten the BTBT window. Under external electric field as well as vertical and biaxial strain, the Ga_2_O_3_/SiC vdW hetero-bilayer maintains the type-III band alignment, revealing its capability to tolerate the external electric field and strain with resilience. All these results provide a compelling platform of the Ga_2_O_3_/SiC vdW bilayer to design high performance tunneling field effect transistor.

## Introduction

Van der Waals (vdW) bilayer heterostructure, comprising two different atomically thin layers of two-dimensional (2D) materials stacked vertically, has attracted significant research attention^1–7^. The remarkable potential of heterostructures to effectively integrate diverse 2D materials at the atomic level renders them an ideal foundation for an extensive range of electrical and optoelectronic applications^8–15^. In contrast to strong covalent bonds within a layer, weak vdW forces remain between layers of the vdW heterostructures, allowing for different stacking configurations of the heterostructures^16,17^. An important characteristic of these heterostructures is their band arrangement, and based on the band alignment, the vdW heterojunctions can be categorized into three types: type-I, type-II, and type-III^18^. Type-I is the straddling band alignment that confines the movement of the electrons and holes spatially to achieve effective recombination of the carriers and, thus, an ideal platform for optical devices, including light-emitting-diodes and lasers^19–21^. On the other hand, type-II is the staggered band alignment that permits the effective separation of the electrons and holes in two different layers; indicating its potential application in photocatalysis, excitonic solar cell and photo-electric device^22–26^. Type-III is the broken gap band alignment where the valence band and conduction band of the heterostructure overlap. In a type-III heterostructure, the conduction band minimum (CBM) resides below the valence band maximum (VBM), enabling the charges to tunnel from the VBM of one layer to the CBM of another layer^27^. This procedure is referred as the band-to-band tunneling (BTBT) effect. The BTBT effect improves the tunneling efficiency and induces negative differential resistance (NDR) effect^28^. Hence, type-III heterostructures are favorable for application in tunneling field-effect transistor and photo-detector^29^. Due to the diversified application prospects of the vdW heterojunctions, intensive research has been carried out in this field. But, most of the reported vdW heterostructures exhibit type-I or type-II band arrangement and very few of the heterostructures are reported to possess type-III band orientation, limiting their application in tunneling field effect transistor (TFET).

Of late, 2D vdW ferroelectric (FE) heterostructures show great potential in electronic and opto-electronic applications attributed to their ultra-thin atomic structure as well as dangling bonds free surface^30–33^. The FE heterostructures can be experimentally fabricated through mechanical exfoliation (ME), metal–organic chemical vapor deposition or self-assembly process^34–36^. Recently, heterostructures based on FE In_2_Se_3_ are reported to show broad optical response, transition in the band alignments, as well as variable Schottky barrier height under polarization reversal, which enhances their use in photoelectric device and FET^30,33,37–39^. FE In_2_Se_3_ monolayer has been reported to realize by vdW epitaxy and mechanical exfoliation^40,41^. Monolayer FE-ZB’ gallium oxide (Ga_2_O_3_) possesses similar atomic architecture as that of In_2_Se_3_ monolayer^42^. Liao et al. reported outstanding dynamic and thermal stability of the Ga_2_O_3_ monolayer through molecular dynamic simulation^42^. Robust ferroelectricity and wide band gap of the Ga_2_O_3_ monolayer have made the material a potential candidate for ferroelectric and opto-electronic applications^42^. In addition, strain and external electric field can be used to tune the electronic property of the 2D Ga_2_O_3_^42,43^. Layered Ga_2_O_3_ is a promising candidate for room temperature nitric-oxide gas sensor^43^. Different polarization states of the Ga_2_O_3_ monolayer result in type-I and type-II band alignment in VS_2_/Ga_2_O_3_ heterostructure and Ohmic and Schottky contact in Gr/Ga_2_O_3_ vdW bilayer^44,45^. Nevertheless, limited studies have been conducted on the broken gap band alignment of Ga_2_O_3_ based heterostructure.

Besides, monolayer SiC is a wide band gap material with stable planar architecture, excellent electrical conductivity, outstanding optical properties, and higher thermal capability^46–49^. The lattice constant of 2D SiC is close to that of FE Ga_2_O_3_ monolayer. It has a honeycomb structure, similar to graphene^48^. The unique properties of the layered SiC have drawn significant interest in device application^50^. Higher break-down field, mechanical strength, as well as in-plane stiffness have made the material an appealing candidate for TFET application^51–53^. Recently, Polley et al. reported the large area bottom-up hetero-epitaxial growth of SiC monolayer^54^. Layered SiC has been combined with other 2D materials including Sn, AlN, and Hf_2_CO_2,_ to adapt heterostructure for electronic device applications^2,55,56^. In this context, Hrubišák et al. recently reported the growth of Ga_2_O_3_ thin films on 4H-SiC substrate by metal–organic chemical vapor deposition method^57^. However, to our knowledge, no study has been conducted on the vdW heterostructure based on layered Ga_2_O_3_ and SiC to reveal its potential in TFET application.

In this article, we report a novel Ga_2_O_3_/SiC vdW heterostructure with type-III band alignment, promising for TFET application by means of first-principles calculation. The underlying physical mechanism for the broken gap alignment has been investigated. The VBM of the SiC layer lies above the CBM of the Ga_2_O_3_ layer which allows the electrons to tunnel, facilitating the BTBT scheme. The Ga_2_O_3_/SiC vdW bilayer has a tunneling window of 0.609 eV and a tunneling probability of 37.83%. We have also analyzed the influence of external electric field, and vertical and biaxial strain on the electric property of Ga_2_O_3_/SiC vdW heterostructure. Positive electric field and compressive vertical strain enhances the BTBT effect due to increased charge transfer from SiC layer to the Ga_2_O_3_ layer. Under the change of these external factors, the hetero-bilayer retains the broken gap alignment, implying its robustness to the external factor. Another type of stacking model, where SiC layer is stacked below the downward polarization sate of Ga_2_O_3_ layer (Ga_2_O_3_$\downarrow$/SiC) exhibits semiconducting property with a type-II band arrangement. These results suggest that the Ga_2_O_3_/SiC heterogeneous bilayer has great promise for tunneling field-effect transistor as well as multipurpose electronic device application.

## Computational methods

First-principles Density Functional Theory (DFT) calculations have been carried out using the Vienna Ab initio Simulation Package (VASP) in the MedeA software setting^58^. To approximate the exchange–correlation potential, we adopted the Perdew–Burke–Ernzerhof (PBE) functional within the generalized gradient approximation (GGA), while projector-augmented wave (PAW) method is employed to define the electron-nuclear interactions^59,60^. We adopted a vacuum region of around 25 Å along the Z axis to exclude the interaction between the two succeeding layers. The pane-wave cut-off energy was taken as 500 eV. We utilized a 12 $\times$ 12 $\times$ 1 Monkhorst–Pack (MP) k-point grid for geometry optimization and electronic band structure calculation, while a 24 $\times$ 24 $\times$ 1 MP grid is used to calculate the density of states (DOS)^61^. We performed Grimme’s DFT-D3 method for the van der Waals correction^62^. A force convergence criterion of 0.01 eV/Å was chosen to optimize the structures while 10^–5^ eV was the energy converge criterion for the self-consistent field. In addition, Density Functional Perturbation Theory (DFPT) is used to compute the dynamic stability forecasting phonon dispersion relation^63^. Charge redistribution in the heterostructure is quantitatively analyzed using Bader charge analysis^64^.

## Results and discussion

**Figure 1:**
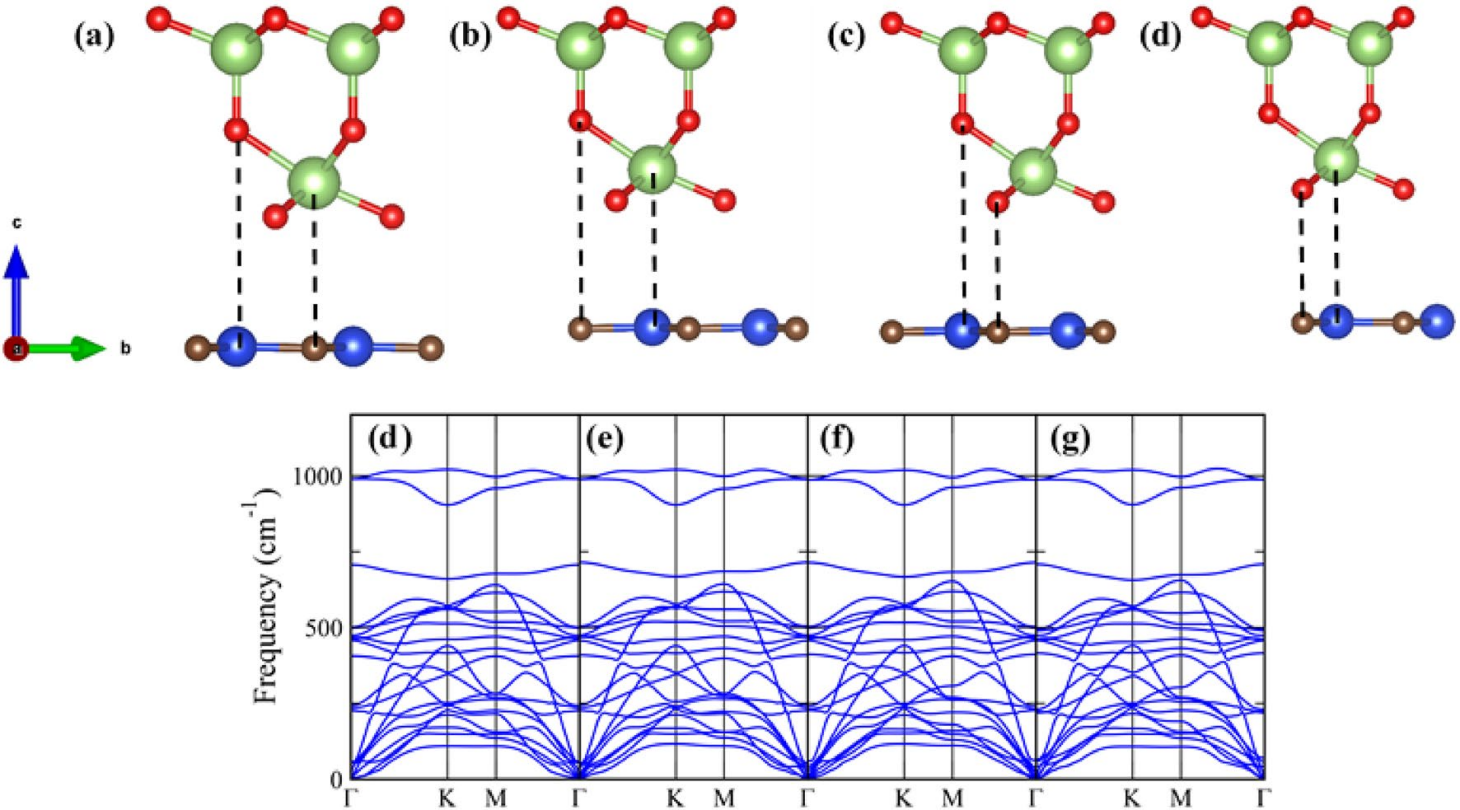
Side view of the four-stacking pattern of the Ga_2_O_3_/SiC vdW bilayer hetero-structure: (**a**) X_1_, (**b**) X_2_, (**c**) X_3_, and (**d**) X_4_. Phonon dispersion relations of the four stacking configurations: (**d**) X_1_, (**e**) X_2_, (**f**) X_3_, and (**g**) X_4_.

The Ga_2_O_3_/SiC van der Waals (vdW) heterostructure is obtained by vertically stacking the SiC layer and Ga_2_O_3_ layer. The structural, electronic property, and electron localization function (ELF) of the individual Ga_2_O_3_ and SiC 2D structures are provided in the supporting information. The lattice mismatch ($${\Delta }$$) between the two layers is 0.08%, as obtained by the relation, $${\Delta } = \frac{{a_{SiC} - a_{{Ga_{2} O_{3} }} }}{{a_{SiC} + a_{{Ga_{2} O_{3} }} }} \times 100\%$$, where $$a_{SiC} $$ and $$a_{{Ga_{2} O_{3} }}$$ are the optimized lattice constants of the SiC and Ga_2_O_3_ monolayer, respectively. The small lattice mismatch between the two layers suggest the experimental realization of the Ga_2_O_3_/SiC vdW heterostructure. Our Ga_2_O_3_/SiC vdW bilayer is comparable to the VS_2_/Ga_2_O_3_ heterostructure where the lattice mismatch between the VS_2_ and Ga_2_O_3_ layer is 2.17%^44^. In addition, Chen et al. studied the photocatalytic and electrical properties of the MoS_2_/Ga_2_O_3_ bilayer where the lattice mismatch between the two layers was 3.31%^39^. Depending on the relative places of the different atoms of the 2D Ga_2_O_3_ and SiC, four stacking patterns are considered, namely as X_1_, X_2_, X_3_, and X_4_, as shown in Fig. 1. The dynamic stability of the Ga_2_O_3_/SiC heterogeneous bilayer is evaluated by calculating the phonon dispersions of the bilayer. The phonon dispersion spectra of the X_1_, X_2_, X_3_ and X_4_ patterns are also illustrated in Fig. 1. The stacking patterns considered are dynamically stable since there is no soft frequency mode present in their phonon dispersion spectra.

The energetic stability of a bilayer structure can be assessed by obtaining the binding energy of the structure. Negative binding energy value suggests the energetic stability of the bilayer structure. The binding energy ($E_{B}$) of the different stacking arrangements of the heterogeneous bilayer is obtained through the relation:

1 $$ E_{B} = \frac{{E_{{{\text{Ga}}_{2} {\text{O}}_{3} /{\text{SiC}}}} - E_{{{\text{Ga}}_{2} {\text{O}}_{3} }} - E_{{\text{SiC }}} }}{S} $$

where $S$ denotes the interface contact area of the heterogeneous bilayer, $E_{{{\text{Ga}}_{2} {\text{O}}_{3} /{\text{SiC}}}}$ represents total energy of the Ga_2_O_3_/SiC hetero-bilayer and $E_{{{\text{Ga}}_{2} {\text{O}}_{3} }}$, $E_{{\text{SiC }}}$ correspond to the energy of the free-standing Ga_2_O_3_ and SiC monolayer, respectively. The binding energies of the stacking models are summarized in Table S1, ranging from − 20.48 to − 28.15 meV/Å^2^. The results suggest that all four models are energetically stable. Table S1 also enlists the optimized interlayer distances of the stacking patterns of the Ga_2_O_3_/SiC heterogeneous bilayer, extending from 3.025 to 3.546Å. The electronic band structures of the stacking models are provided in Figure S2. As Figure S2 indicates, the electronic band diagrams of the four models show metallic properties, with the VBM is above the Fermi level and the CBM is below the Fermi level. This indicates that all the stacking patterns of the Ga_2_O_3_/SiC bilayer possess type-III (broken gap) band alignment.

**Figure 2:**
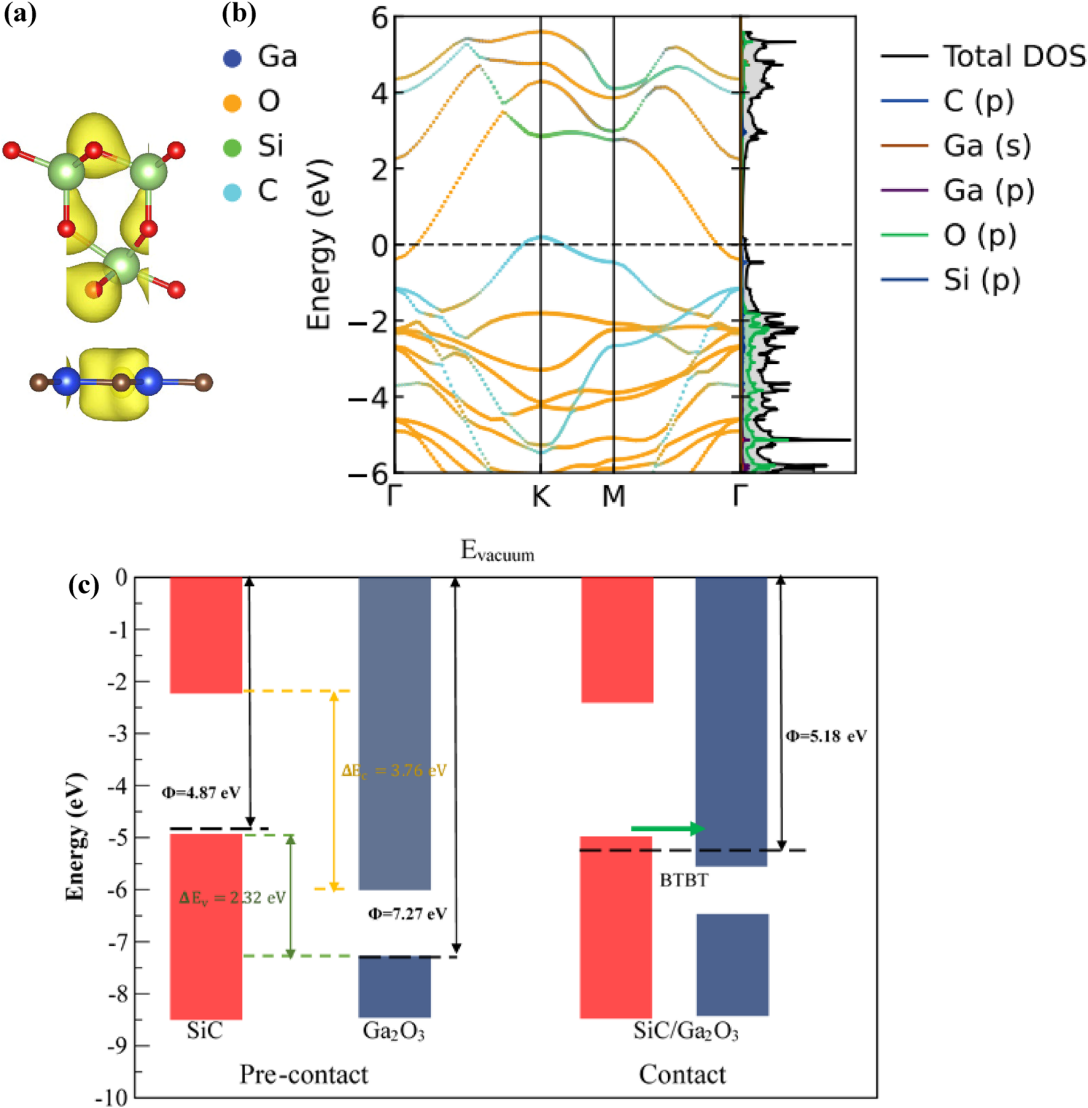
(**a**) Electron localization function, (**b**) projected band structure and partial density of states (PDOS) of the Ga_2_O_3_/SiC vdW bilayer heterostructure. The Fermi level is set to the zero value. In the projected band diagram, the contribution from the Ga, O, Si, and C atom is represented by the navy blue, cyber-yellow, green, and sky-blue colors, respectively. (**c**) Band alignment of the Ga_2_O_3_ and SiC layer before and after forming the Ga_2_O_3_/SiC vdW bilayer heterostructure. ${\Phi }$ refers to the work-function of the material.

Among the four (dynamic stable) models, pattern X_1_ is the most energetically favorable model with the minimum binding energy (− 28.15 meV/Å^2^) and optimized interlayer distance (3.025 Å). Hence, for our subsequent study, we focus on the X_1_ stacking model of the Ga_2_O_3_/SiC heterogeneous bilayer. The ELF of the Ga_2_O_3_/SiC bilayer is illustrated in Fig. 2a, where from weak interlayer interaction between the two layers can be visualized. No electron is localized at the hetero-bilayer interface, suggesting the absence of binding between the Ga_2_O_3_ and SiC layer and verifying weak vdW interaction between the two layers of the Ga_2_O_3_/SiC bilayer.

Next, we study the type of band alignment, which is an important intrinsic property of the vdW bilayer heterostructure. The projected band structure and the partial density of states (PDOS) of the X_1_ model of the Ga_2_O_3_/SiC bilayer structure are represented in Fig. 2b. The Fermi energy level is set to the zero value. The contribution of the Ga, O, Si and C atom in the electronic band diagram is denoted by the navy blue, cyber yellow, green and sky-blue colors, respectively. Apparently, VBM comes from the C atom of the SiC layer and is located on the K point. CBM is contributed by the O atom of the of the Ga_2_O_3_ layer and located at the ${\Gamma }$ point. It can be clearly observed that CBM lies below the VBM, generating a negative band gap of 0.609 eV and thus confirming a type-III (broken gap) band alignment of the Ga_2_O_3_/SiC bilayer structure. It is important to note that, these type-III vdW heterogeneous bilayers exhibit apparent negative differential resistance characteristics owing to the presence of band-to-band tunneling (BTBT)^65^.

To shed light on the physical mechanism of the broken gap band arrangement of the Ga_2_O_3_/SiC heterogeneous bilayer, schematic illustration of the band alignments of the free-standing Ga_2_O_3_ monolayer, free-standing SiC monolayer and the Ga_2_O_3_/SiC vdW bilayer are shown in Fig. 2c. Work function (${\Phi }$) serves as an inherent reference for the band arrangement and can be obtained employing the relation:

2 $$ {\Phi } = E_{vacuum} - E_{Fermi} $$

where $E_{vacuum}$ corresponds to stationary electron’s energy level adjacent to the surface and $E_{Fermi}$ denotes the Fermi energy level. The difference in the energy of the CBM between the Ga_2_O_3_ and the SiC monolayer (${\Delta }E_{c}$) is 3.76 eV while the energy difference in the VBM (${\Delta }E_{v}$) is 2.32 eV. For the freestanding SiC and Ga_2_O_3_ monolayers, as shown in Fig. 2c, the calculated work functions are 4.87 eV and 7.27 eV, respectively. Notably, the VBM of pristine SiC is higher than the CBM of the pristine Ga_2_O_3_ layer. Hence, when the two separate layers come in contact to build the bilayer heterostructure, electrons from the SiC monolayer will move naturally to the Ga_2_O_3_ layer to align the Fermi energy level and reach the same work function all through the bilayer heterostructure. The work function value of the Ga_2_O_3_/SiC hetero-bilayer, as we obtained is 5.18 eV, which is between the work function values of the free-standing SiC and Ga_2_O_3_ layer. Once the charge transfer is complete, the Fermi energy level shifts upward to become slightly higher than the CBM of the Ga_2_O_3_ layer while it shifts downward to become slightly lower than the VBM of the SiC layer. (All together, the VBM of the SiC layer also shifts slightly downward). Consequently, the vdW hetero-bilayer harbors a broken-gap band arrangement and the electrons can tunnel from the VBM of the SiC layer below the Fermi energy level to the CBM of the Ga_2_O_3_ layer above the Fermi energy level owing to the type-III band alignment feature. This is called the BTBT phenomenon, occurring in the type-III vdW heterogeneous bilayer and suggesting its potential application in tunneling field effect transistor (TFET).

**Figure 3:**
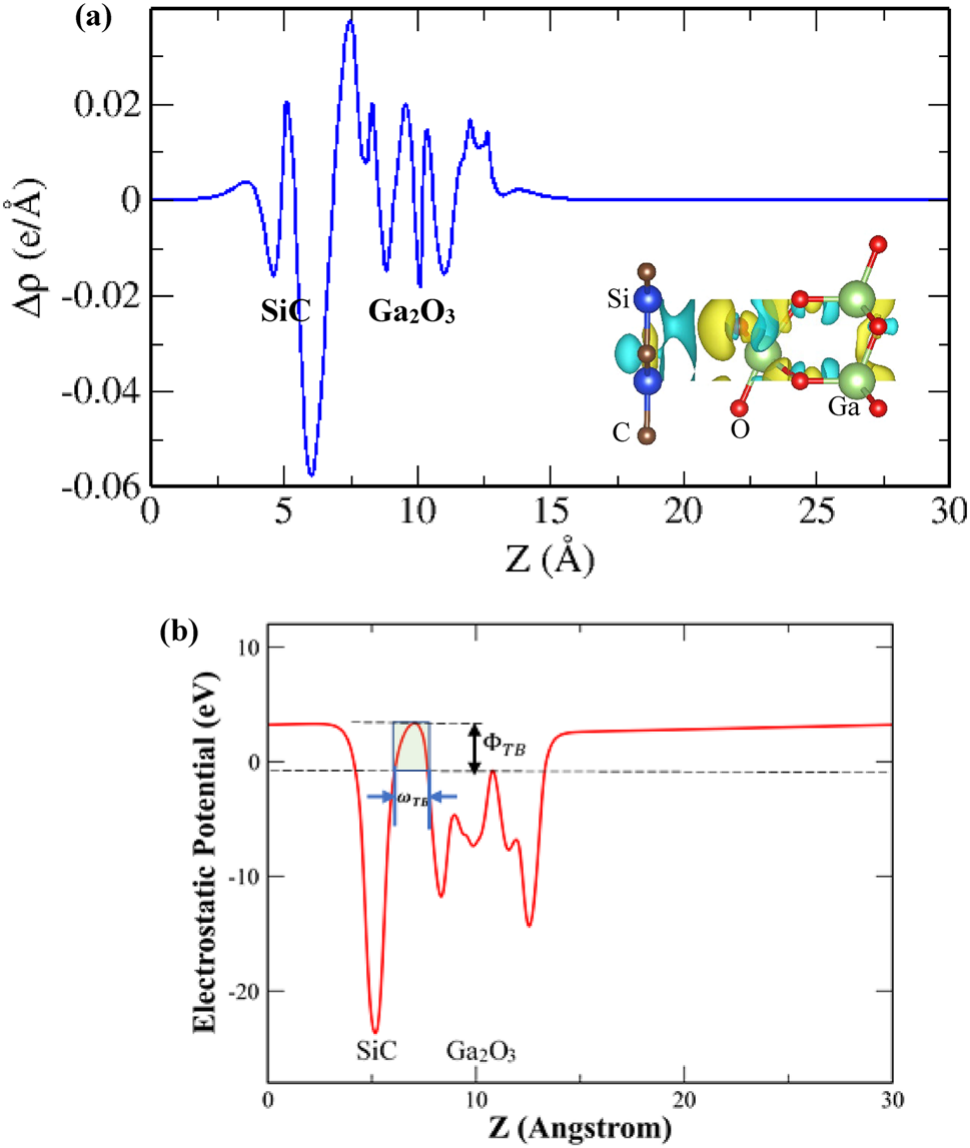
(**a**) Plane-averaged charge density difference of the Ga_2_O_3_/SiC vdW bilayer heterostructure along the Z axis. The inset shows the 3D iso-surface of the charge density difference of the Ga_2_O_3_/SiC vdW bilayer heterostructure with an iso-value of 0.0005 e/ Å^3^. The yellow and cyan colors represent the accumulation and depletion of charge, respectively. (**b**) Electrostatic potential profile of the Ga_2_O_3_/SiC vdW bilayer heterostructure.

In order to reveal the charge rearrangement and the formation mechanism of the interlayer coupling of the Ga_2_O_3_/SiC heterogeneous structure, we calculated the plane-averaged charge density difference along the Z-axis as well as the three-dimensional iso-surface of the charge density difference of the heterostructure. The charge density difference of the vdW heterostructure is obtained employing the following relation:

3 $$ \Delta \rho = \rho_{{{\text{Ga}}_{2} {\text{O}}_{3} /{\text{SiC}}}} - \rho_{{{\text{Ga}}_{2} {\text{O}}_{3} }} - \rho_{{{\text{SiC}}}} $$

where $\Delta \rho$ is the charge density difference of the Ga_2_O_3_/SiC heterogeneous structure and $\rho_{{{\text{Ga}}_{2} {\text{O}}_{3} /{\text{SiC}}}}$, $\rho_{{{\text{Ga}}_{2} {\text{O}}_{3} }}$ and $\rho_{{{\text{SiC}}}}$ denote the charge density of the bilayer heterostructure, free-standing Ga_2_O_3_ layer and free-standing SiC layer, respectively. The plane-averaged charge density difference of the vdW bilayer is represented in Fig. 3a while the inset shows the 3D iso-surface of the charge density difference. The yellow color represents an increase of charge while the cyan color represents a decrease of charge. From Fig. 3a, one can clearly observe that, charge is depleted mostly from the SiC layer while it is accumulated on the Ga_2_O_3_ layer, suggesting the transfer of charge from the SiC layer to the Ga_2_O_3_ layer. Charge redistribution mainly occurred at the interface region of the vdW heterogeneous bilayer. In addition, Bader charge analysis is carried out to quantify the charge redistribution at the heterostructure interface which reveals that an amount of 0.079e charge is transferred from the SiC layer to the Ga_2_O_3_ layer. This confirms that a built-in electric field is induced at the heterostructure interface and supports the formation mechanism stated earlier.

An important factor for the field effect transistor performance is the tunneling probability, which can be calculated from the energy barrier at the heterostructure interface. A tunneling barrier is generated by the accumulation of negative charges at the heterostructure interface. This tunneling barrier can be quantitatively illustrated in the electrostatic potential diagram, as seen in Fig. 3b ^66,67^. The tunneling barrier is reduced by the type-III broken gap, resulting in a significant tunneling probability^68,69^. The following relation is utilized to calculate the tunneling probability^70^:

4 $$ P_{TB} = exp\left( { - \frac{{\omega_{TB} }}{\hbar }\sqrt {m{\Phi }_{TB} } } \right) $$

where $P_{TB}$ is the tunneling probability, $\hbar$ is the reduced Planck constant and $m$ is the free electron mass. $\omega_{TB}$ and ${\Phi }_{TB}$ refer to the tunneling barrier width and height, respectively and illustrated in Fig. 3b. ${\Phi }_{TB}$ is the difference in height between the maximum potentials of the vdW space and the Ga_2_O_3_ monolayer while $\omega_{TB}$ is described as the vdW space barrier width at the maximum potential of the Ga_2_O_3_ monolayer. In our calculation, we obtained 4.19 eV and 1.61 Å as the tunneling barrier height and width, respectively and the tunneling probability is 37.83%. This is higher than the tunneling probability of the InS/Au bilayer structure^71^ as well as that of the KAgSe/ SiC_2_ vdW heterogeneous bilayer^72^ and suggests a higher efficiency in the injection of the carriers for the Ga_2_O_3_/SiC hetero-bilayer.

Charge transfer across the interface of the vdW heterogeneous bilayer substantially affect the electronic properties of the hetero-bilayer, and various external factors, including biaxial strain, external electric field, as well as vertical strain can efficiently tune the electronic properties of the vdW hetero-bilayer^15,73–81^. Hence, the electronic properties of the Ga_2_O_3_/SiC bilayer are systematically studied under the influence of these external factors.

**Figure 4:**
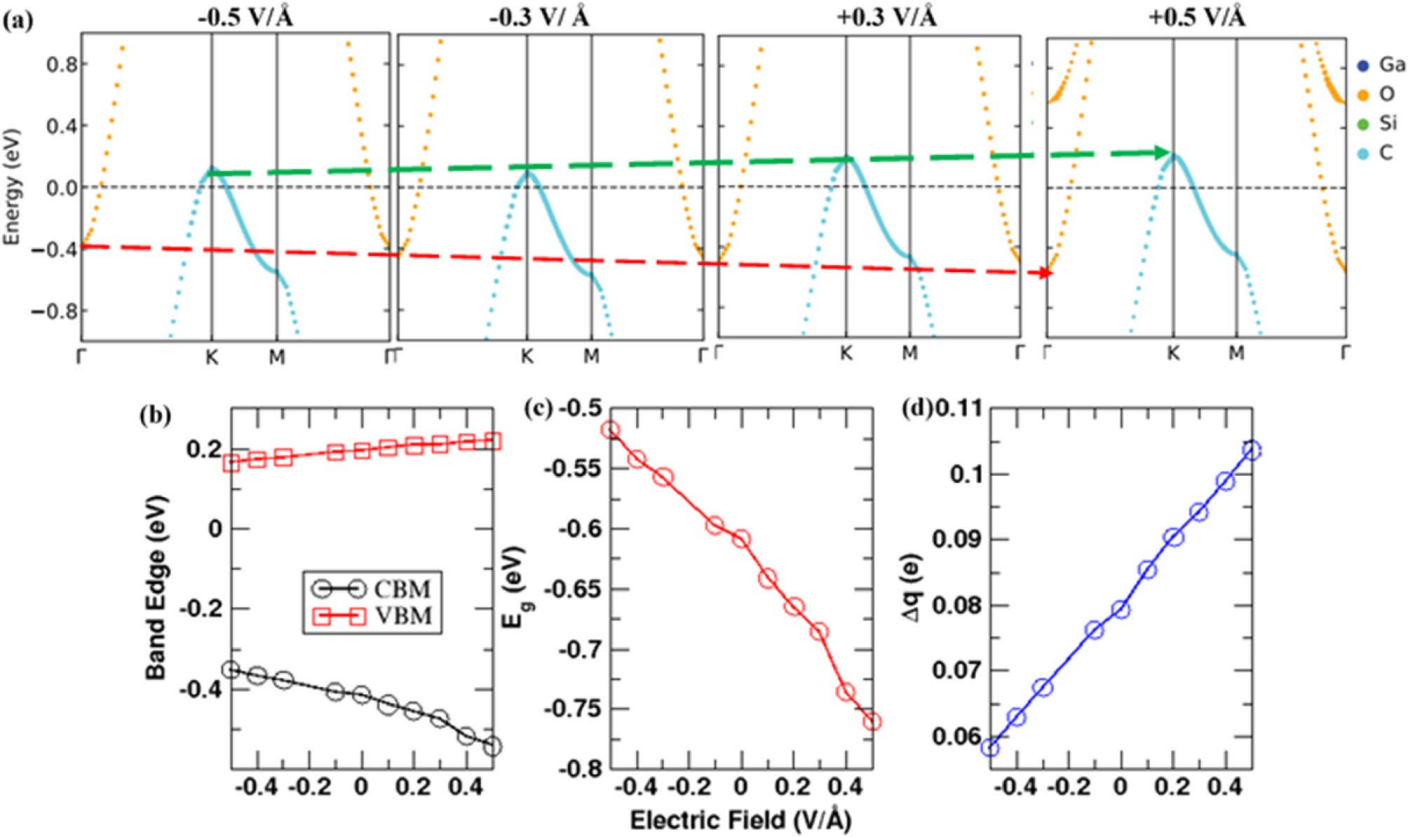
(**a**) Projected band diagram of the Ga_2_O_3_/SiC vdW bilayer heterostructure under different electric fields applied. (**b**) The change of CBM and VBM, (**c**) electronic band gap (E_g_), and (**d**) charge transfer (${\Delta q}$) of the Ga_2_O_3_/SiC vdW bilayer heterostructure as a function of external electric field applied along the Z axis.

In practical applications, the electronic behaviors of the two-dimensional materials and devices can be tuned by the gate voltage to address various application requirements^82^. Therefore, the influence of the gate voltage on the electronic behavior of the Ga_2_O_3_/SiC vdW hetero-bilayer is examined by applying an electric field perpendicular to the layers of the heterostructure (along the Z axis). Herein, the positive electric field points from the SiC layer to the Ga_2_O_3_ layer while the negative electric field points from the Ga_2_O_3_ layer to the SiC layer. The atom projected electronic band structures of the Ga_2_O_3_/SiC layer under electric fields ranging from − 0.5 to + 0.5 V/Å are illustrated in Fig. 4a. Figure 4b,c present the band edge positions and electronic band gap (E_g_) under the variation of the external electric field, respectively. The broken-gap band alignment feature is well maintained within this range of external electric field. Under a positive electric field, the CBM of the bilayer contributed by the Ga_2_O_3_ layer goes downward (indicated by the dashed red arrow in Fig. 4a), while the bilayer VBM contributed by the SiC layer is pushed upward (indicated by the dashed green arrow in Fig. 4a), hence increasing the magnitude of the negative electronic band gap (Fig. 4c). Alternatively, the tunneling energy window, which is the difference in energy from the VBM of the SiC layer to the CBM of the Ga_2_O_3_ layer, is expanded upon the application of the positive electric field. Accordingly, there will be an increase in the quantity of electron tunneling from the VBM of the SiC layer to the Ga_2_O_3_ layer CBM. Thus, the tunneling probability will be increased, which will enhance the BTBT mechanism. On the other hand, applying an external negative electric field causes the VBM of the Ga_2_O_3_/SiC bilayer heterostructure to shift downward while the CBM of the hetero-bilayer moves upward (Fig. 4a,b), thereby decreasing the magnitude of the negative band gap. Consequently, the tunneling window is narrowed, and this will decrease the amount of charge tunneling from the SiC layer VBM to the Ga_2_O_3_ layer CBM. Thus, tunneling probability is reduced under the influence of a negative electric field and the BTBT mechanism is weakened. A similar type of phenomenon is also reported in the WTe_2_/HfS_2_ vdW heterostructure studied by Lei et al. ^83^.

Analysis on the direction of the applied external electric field and the intrinsic built-in electric field at the interface of the Ga_2_O_3_/SiC vdW bilayer provides an intuitive understanding of the evolution of the electronic band structure of the vdW hetero-bilayer upon the application of external electric field. The amount of charge transfer (${\Delta q}$) with respect to the applied electric field is summarized in Fig. 4d. The direction of the positive external electric field coincides with the inherently built-in electric field of the hetero-bilayer. Therefore, applying positive external electric field reinforces the inherent built-in electric field and the amount of charge transfer from SiC layer to the Ga_2_O_3_ layer is significantly increased, as illustrated in Fig. 4d. This leads to an upshift in the VBM of the vdW bilayer and a downshift in the heterogeneous bilayer CBM, resulting in the tunneling window to broaden and an enhancement in the BTBT mechanism. On the other hand, negative external electric field is directed opposite to the direction of built-in electric field. Hence, negative external electric field weakens the built-in electric field and the amount of electron transfer at the interface is decreased (Fig. 4d). As a result, the tunneling energy window is reduced and the VBM moves downward while the CBM shifts upward. Moreover, we have also studied the effect of external electric fields on the structure of the Ga_2_O_3_/SiC vdW bilayer heterostructures, which is provided in the supporting information.

**Figure 5:**
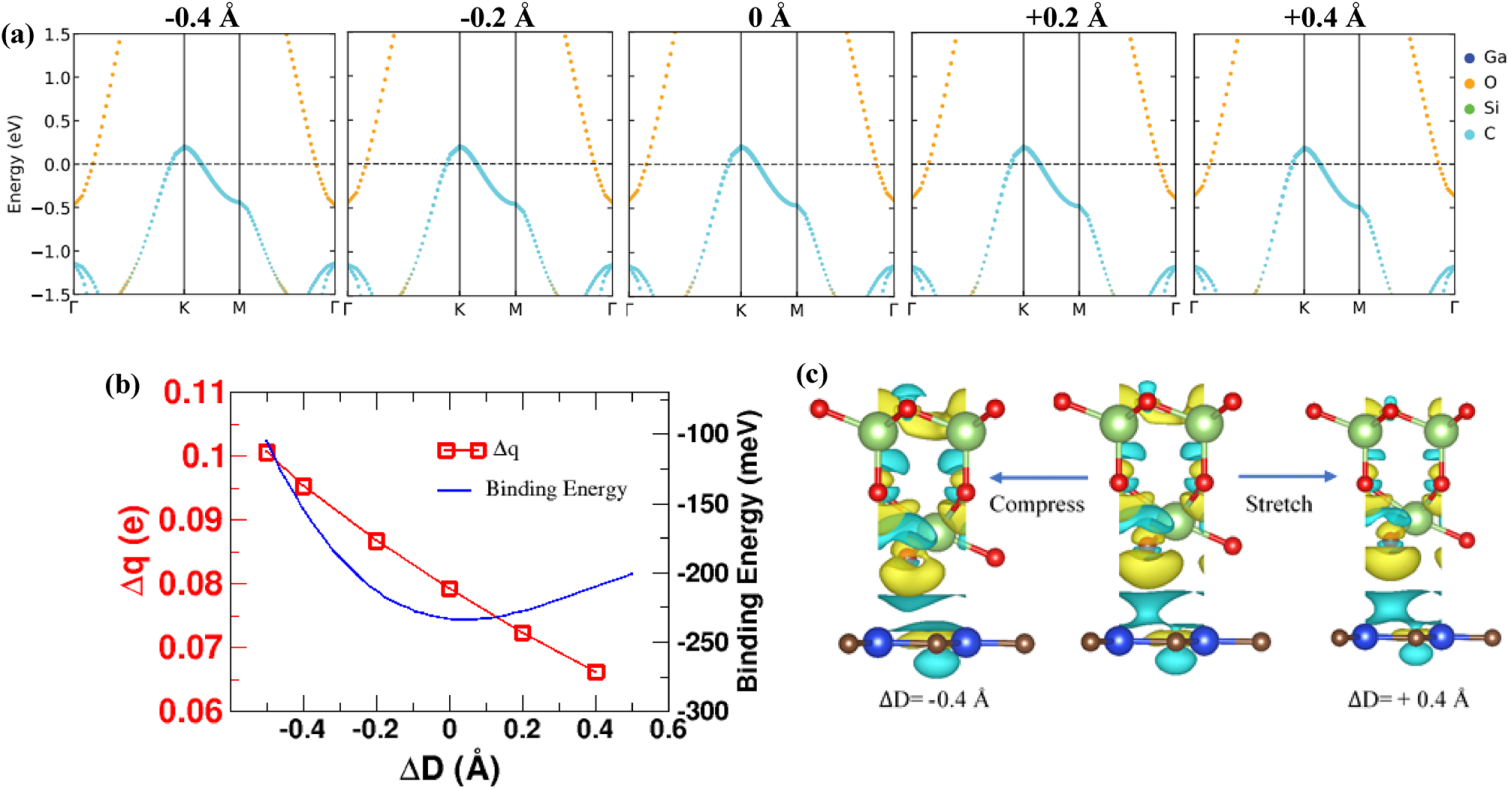
(**a**) Projected band diagram of the Ga_2_O_3_/SiC vdW bilayer heterostructure under various vertical strains applied to the heterostructure. The Fermi level is set to the zero value. (**b**) The change of the binding energy and charge transfer (${\Delta q}$) of the bilayer heterostructure with respect to the vertical strain applied to it. (**c**) 3D iso-surface of the charge density difference of the Ga_2_O_3_/SiC vdW bilayer heterostructure under different vertical strains applied to it. The cyan and the yellow colors denote the decrease and increase of charge, respectively.

Strain engineering is regarded as an effective approach to trigger the electronic properties of the two-dimensional materials^84,85^. Herein, the effect of vertical strain on the electronic properties of the Ga_2_O_3_/SiC vdW hetero-bilayer is studied. Vertical strain (${\Delta D}$) is applied to the heterostructure through changing its interlayer distance. We introduced vertical strain (${\Delta D}$) ranging from − 0.5 to + 0.4 Å to the Ga_2_O_3_/SiC vdW heterostructure. A positive value of ${\Delta D}$ corresponds to tensile vertical strain while a negative value is for compressive vertical strain. Experimentally, vacuum thermal annealing or applying pressure using a scanning tunneling microscope tip can be used to modify the interlayer distance^86,87^. The evolution of the band structure under various vertical strain is shown in Fig. 5a. The electronic band diagram of the Ga_2_O_3_/SiC vdW bilayer maintains the type-III band alignment at different interlayer distances, as shown in Fig. 5a. This suggests that Ga_2_O_3_/SiC vdW bilayer's distinct band alignment can firmly tolerate vertical strain. Besides, applying compressive vertical strain causes the VBM of the SiC layer to rise while the CBM from the Ga_2_O_3_ layer moves downward. This increases the magnitude of the negative band gap (Figure S4) and widens the tunneling energy window, and the BTBT effect is increased. Conversely, when tensile vertical strain is applied to the heterostructure, the SiC layer VBM pulls down at the ${\Gamma }$ point while the CBM of the Ga_2_O_3_ layer at the K point rises, leading to the magnitude of the E_g_ to decrease (Figure S4) and hence narrowing the tunneling energy window.

To shed light on the effect of vertical strain on the electronic behavior of the Ga_2_O_3_/SiC bilayer heterostructure, the amount of charge transfer and the binding energy change under varying vertical strain is depicted in Fig. 5b. The binding energy of the vdW bilayer is negative under all vertical strains considered, suggesting the energetic stability of the system regardless of the vertical strain applied. Besides, when the system is without vertical strain (${\Delta D}$ = 0), the negative binding energy is the maximum, agrees well with earlier study^88^. With the increase of the interlayer distant of the Ga_2_O_3_/SiC bilayer system, the amount of charges transferred (${\Delta q}$) decreases monotonically, revealing weaker interlayer interaction while the interlayer distant is increased. 3D iso-surface of the charge density difference plot of the Ga_2_O_3_/SiC bilayer under ${\Delta D}$ = − 0.4 Å, 0 Å, and + 0.4 Å is shown in Fig. 5c, wherefrom it can be clearly visualized that, reducing the interlayer distant of the bilayer system causes increased charge redistribution at the interface of the Ga_2_O_3_/SiC bilayer.

**Figure 6:**
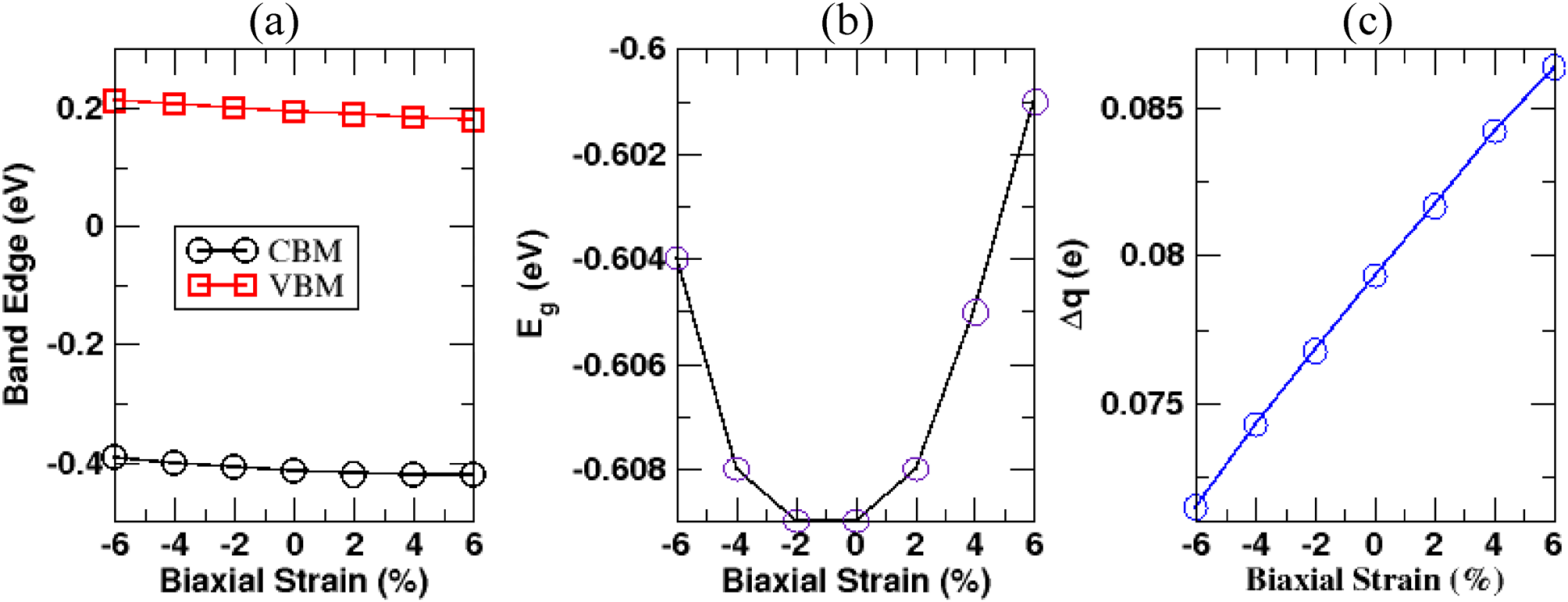
The change of (**a**) CBM and VBM position, (**b**) band gap (E_g_), and (**c**) charge transfer (${\Delta q}$) of the Ga_2_O_3_/SiC vdW bilayer heterostructure under various biaxial strains applied to the heterostructure.

Apart from vertical strain, we also investigate the effect of biaxial strain on the electronic properties of the Ga_2_O_3_/SiC bilayer heterostructure. The biaxial strain has been incorporated to the heterostructure using the following relation:

5 $$ e = \frac{{a - a_{0} }}{{a_{0} }} \times 100\% $$

where $e$ is the percentage of biaxial strain applied to the heterostructure, $a_{0}$ corresponds relaxed lattice constant of the system while a denotes the strained lattice constant of the heterostructure. A positive value of $e$ corresponds to tensile biaxial strain, while a negative value refers to compressive biaxial strain. Biaxial strain ranging from − 6 to + 6% is applied to the heterostructure system. Figure 6 summarizes the results of applying biaxial strain, while the evolution of the band structure under varying percentages of biaxial strain is shown in Figure S5. Compressive biaxial strain causes both the VBM of the SiC layer and CBM of the Ga_2_O_3_ layer to rise while the tensile biaxial strain pushes both the VBM and CBM of the bilayer system downward (Fig. 6a). However, in both cases, the magnitude of the negative band gap (E_g_) decreases slightly from the unstrained band gap (Fig. 6b). There is a slight increase in the amount of charges transfer (${\Delta }$ q) from the SiC layer to the Ga_2_O_3_ layer with the increasing percentage of biaxial strain, as shown in Fig. 6c. Overall, Ga_2_O_3_/SiC bilayer system preserves the type-III band arrangement under varying percentages of biaxial strain, revealing that the bilayer system is capable of withstanding biaxial strain with resilience. In addition, we have also examined the dynamic stability of the Ga_2_O_3_/SiC vdW bilayer heterostructure under biaxial strain by calculating its phonon dispersion relations. The dynamic stability of the bilayer system under biaxial strain from + 6 to − 6% strain is discussed in the supporting information.

**Figure 7:**
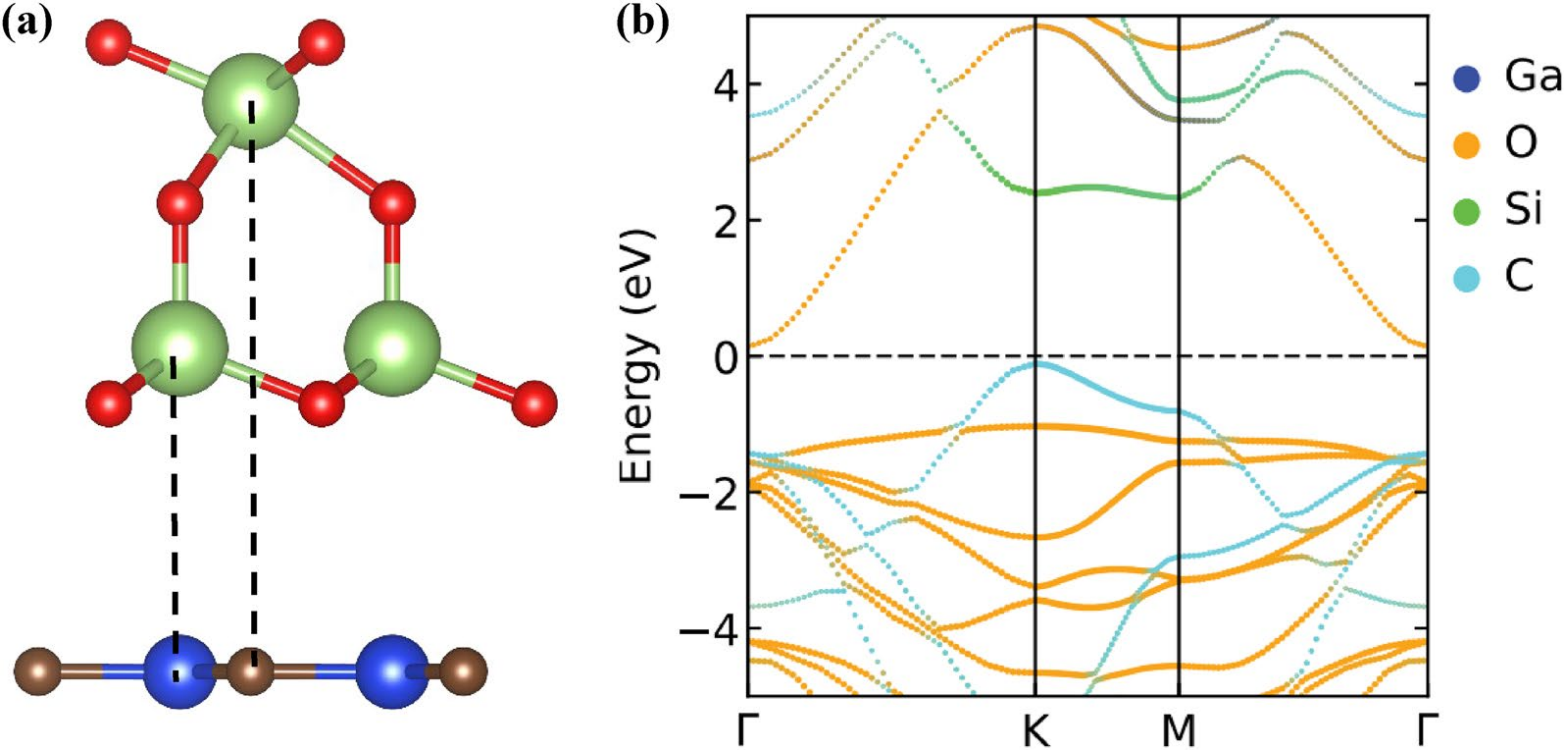
(**a**) Stacking model of the Ga_2_O_3_/SiC vdW bilayer heterostructure considering the downward polarization state of the FE Ga_2_O_3_ layer, and (**b**) the corresponding projected band diagram.

As the FE Ga_2_O_3_ monolayer has two polarization states, there is another polarization state of the Ga_2_O_3_ monolayer, where the middle O atom is near the upper O layer (regarded as downward polarization). Hence, another type of stacking model of the hetero-bilayer is possible when the SiC layer is stacked below the Ga_2_O_3_ layer with its downward polarization state (denoted as Ga_2_O_3_$\downarrow$/SiC). We also investigated the structural and electronic properties of the Ga_2_O_3_$\downarrow$/SiC vdW hetero-bilayer taking a representative stacking model of the Ga_2_O_3_$\downarrow$/SiC vdW heterostructure as shown in Fig. 7a. The optimized interlayer distance is 3.38 Å while the binding energy for the Ga_2_O_3_$\downarrow$/SiC vdW heterostructure system is − 17.23 meV/${\text{ {\AA}}}$^2^, indicating the energetic stability of the system. The atom projected band structure of Ga_2_O_3_$\downarrow$/SiC vdW heterostructure is shown in Fig. 7b. The bilayer system shows a semiconducting property with an electronic gap of 0.243 eV. The VBM is contributed by the C atom of the SiC layer while CBM comes from the O atom of the Ga_2_O_3_ layer, confirming the type-II (staggered) band alignment. Such a type-II band alignment is appealing for solar energy conversion and optoelectronic devices^89^. Therefore, Ga_2_O_3_/SiC vdW heterogeneous bilayer combines the features of the type-II and type-III band alignments when SiC layer is stacked below the Ga_2_O_3_ layer considering its two different polarization states.

## Conclusion

To conclude, a novel Ga_2_O_3_/SiC vdW bilayer heterostructure with inherent type-III broken gap band alignment has been revealed through first-principles DFT calculation. Negative binding energy of the Ga_2_O_3_/SiC bilayer system suggests energetic stability while the dynamic stability of the heterostructure was confirmed by the phonon dispersion spectrum. A tunneling window of 0.609 eV is created due to the overlapping band structures, fulfilling the required condition for the BTBT mechanism, enabling the charges to tunnel from the VBM of the SiC layer to CBM of the Ga_2_O_3_ layer. The effect of external electric field and strain on the electronic properties of the Ga_2_O_3_/SiC vdW hetero-bilayer has been studied. External vertical electric fields and strains can be introduced to adjust the electronic behavior of the bilayer system to enhance the BTBT effect. Positive external electric field and compressive vertical strain widen the tunneling window and enhance the BTBT effect while negative electric field and tensile vertical strain decrease the tunneling window. Under external electric field and different types of strain, the Ga_2_O_3_/SiC vdW bilayer system maintains type-III broken gap band formation, revealing that the system is capable of withstanding external electric field and strain with resilience. Another type of configuration of the bilayer, where the SiC layer is stacked below the downward polarization state of the Ga_2_O_3_ layer, shows a type-II band alignment with an electronic gap of 0.243 eV. All these findings reveal that the Ga_2_O_3_/SiC vdW heterogeneous bilayer offers tremendous potential for tunneling field-effect transistor as well as multi-purpose electronic device.

### Supplementary Information

Supplementary Information.

## Data Availability

The data that support the findings of this study are available from the corresponding author upon reasonable request.
