# Supplementary material for: A resilient type-III broken gap Ga2O3/SiC van der Waals heterogeneous bilayer with band-to-band tunneling effect and tunable electronic property
Source: Sci Rep. 2024 Jun 3;14:12748. doi: 10.1038/s41598-024-63354-8 (PMC11148157; doi:10.1038/s41598-024-63354-8)
Supplement: Supplementary file 1 — Supplementary Information. [file 41598_2024_63354_MOESM1_ESM.docx]

Supporting Information

**A resilient type-III broken gap Ga_2_O_3_/SiC van der Waals heterogeneous bilayer with band-to-band tunneling effect and tunable electronic property**

Naim Ferdous^1^, Md. Sherajul Islam^1,2^*, & Jeongwon Park^1,3^

^1^Department of Electrical & Biomedical Engineering, University of Nevada, Reno, NV 89557, USA.

^2^Department of Electrical and Electronic Engineering, Khulna University of Engineering & Technology, Khulna 9203, Bangladesh.

^3^School of Electrical Engineering and Computer Science, University of Ottawa, Ottawa, ON K1N6N5, Canada.

*Corresponding author. Email: [sheraj_kuet@eee.kuet.ac.bd](mailto:sheraj_kuet@eee.kuet.ac.bd)


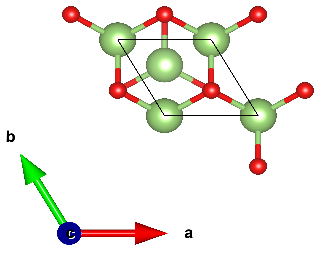

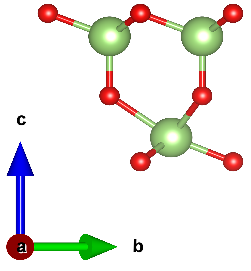

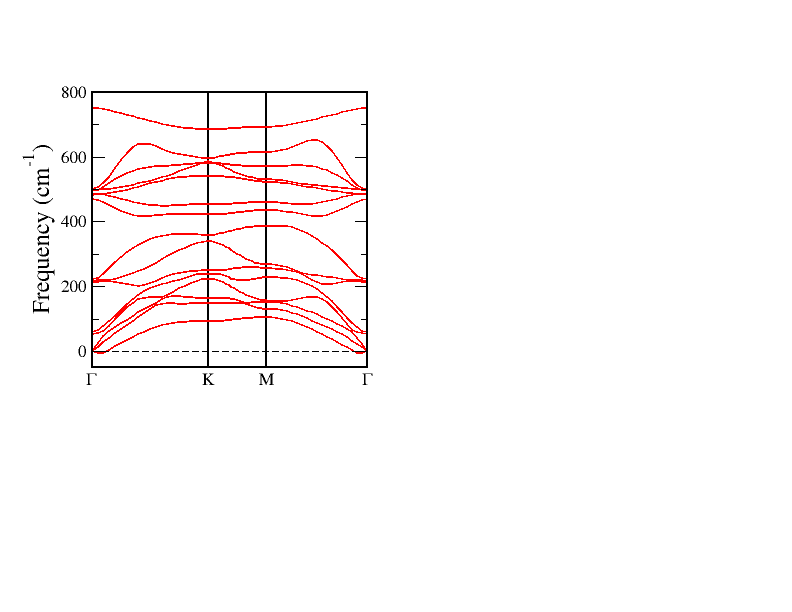

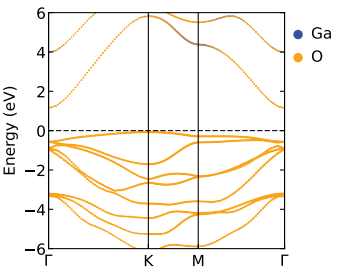

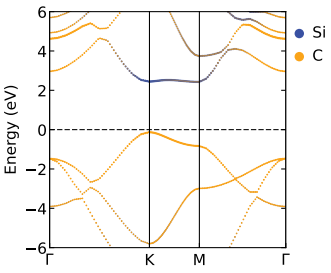

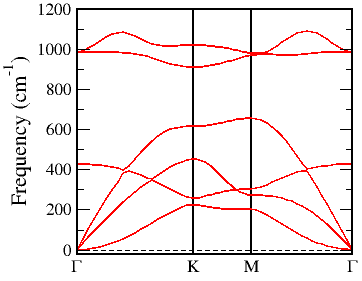

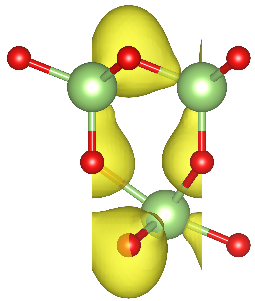

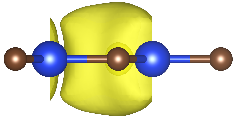


**(a) (b) (c)**

**(d) (e) (f)**

**(g)**


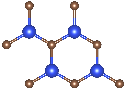


**Figure S1.** (a) Top view and side view of the unit cell of the Ga_2_O_3_ monolayer. (b) Projected band structure of the Ga_2_O_3_ monolayer. The contributions from the Ga and O atom are represented by the navy blue and cyber-yellow colors, respectively. (c) Phonon dispersion relations of the Ga_2_O_3_ monolayer. (d) Electronic band structure of the SiC monolayer. The contributions from the Si and C atom are represented by the navy blue and cyber-yellow colors, respectively. The inset shows the top view of the SiC monolayer. Electron localization function of the (f) Ga_2_O_3_ monolayer, and (g) SiC monolayer. Yellow overlayers represent the localization of the electrons.

The geometry-optimized structure of the Ga_2_O_3_ mono-layer is shown in Figure S1(a). We obtained a lattice constant of 3.09 Å for the geometry-optimized structure of 2D Ga_2_O_3_, consistent with the work of Liao et al^1^. Three O-layers and two Ga-layers alternate in the structure of the FE-Ga_2_O_3_ layer while the movement of the internal O-layer determines the structure’s polarization inversion. We have also calculated the polarization value of the Ga_2_O_3_ monolayer. In our calculation, we obtained the polarization of 2D Ga_2_O_3_ as 14.9 nC/cm^2^. The electronic band diagram of the layered Ga_2_O_3_ is illustrated in Figure S1(b). Layered Ga_2_O_3_ is a semiconductor with an indirect band gap of 1.202 eV. The valence band maximum (VBM) is located at the K point while the conduction band minimum is located at the $\Gamma$ point. In order to verify the dynamic stability of the layered Ga_2_O_3_, the phonon dispersion spectrum of the Ga_2_O_3_ layer is calculated. As Figure S1(c) suggests, soft-frequency phonon mode is absent in the dispersion relation, indicating that monolayer Ga_2_O_3_ is dynamically stable. Figure S1(d) presents the band structure of 2D SiC while the inset shows the top view of the unit cell of 2D SiC. As our calculation yields, the optimized lattice constant of the 2D SiC is 3.095 Å, which agrees well with the earlier studies^2–4^. 2D SiC is semiconductor with an indirect band gap of 2.54 eV, VBM located at the K-point while CBM located at the $\Gamma$ point. The phonon dispersion spectrum of the monolayer 2D SiC (Figure S1(e)) indicates that there is no negative frequency phonon mode, confirming the dynamic stability of the monolayer SiC. Figure S1(f) and (g) depict the electron localization function (ELF) of the layered Ga_2_O_3_ and SiC, respectively, where form the covalent bonding characteristic between the Si and C atoms as well as Ga and O atoms can be observed.


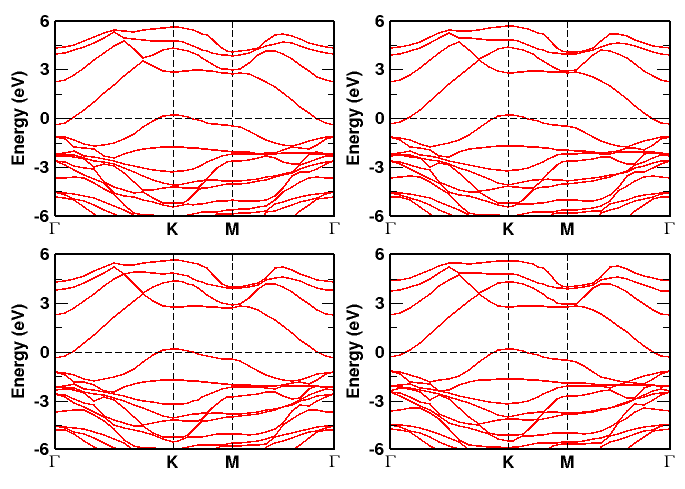


**(a) (b)**

**(c) (d)**

**Figure S2.** Electronic band diagram of the four configurations of the Ga_2_O_3_/SiC vdW bilayer heterostructure. (a) X_1_, (b) X_2_, (c) X_3_, (d) X_4_ configuration.


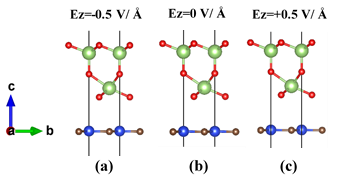


**Figure S3:** Side view of the geometry optimized structure of the Ga_2_O_3_/SiC vdW bilayer heterostructure under different external electric field along the Z axis (E_z_): (a) E_z_ = -0.5 V/Å, (b) E_z_ = 0 V/Å, and (c) E_z_ = +0.5 V/Å.

In order to study the effect of external electric field on the geometric structure of the Ga_2_O_3_/SiC vdW bilayer heterostructure, the relaxed structures of the bilayer system under external electric fields along the Z direction at -0.5 V/Å and +0.5 V/Å are obtained. Figure S3 illustrates the geometry relaxed structures of the Ga_2_O_3_/SiC vdW bilayer heterostructure under different external electric fields. As one can clearly observe, the unit cell of the Ga_2_O_3_/SiC vdW bilayer heterostructure retains its original structure under electric fields at -0.5 V/Å and +0.5 V/Å without any significant distortion observed. Hence, external electric field in the range -0.5 V/Å to +0.5 V/Å rarely affect the geometric structure of the Ga_2_O_3_/SiC vdW bilayer heterostructure and no structural distortion is noticed in the bilayer system.


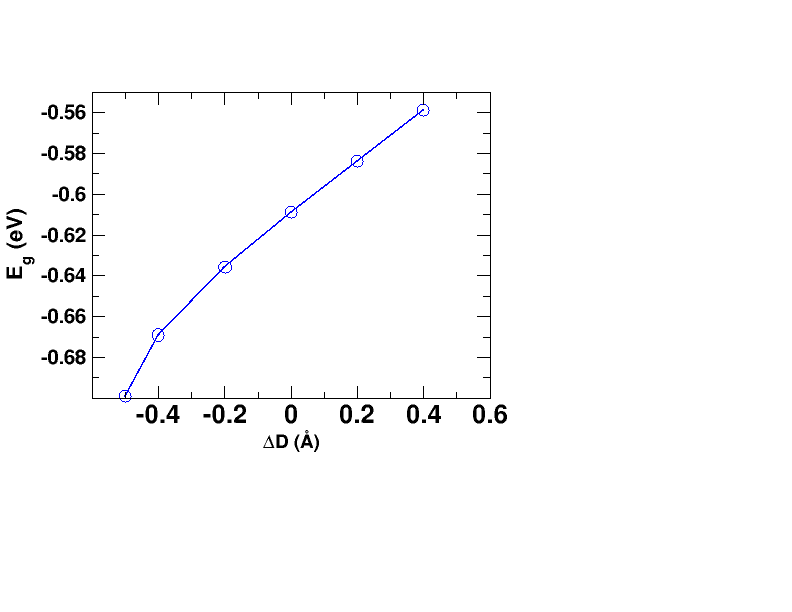


**Figure S4.** The variation of the band gap (E_g_) as a function of vertical strain applied to the Ga_2_O_3_/SiC vdW bilayer heterostructure.


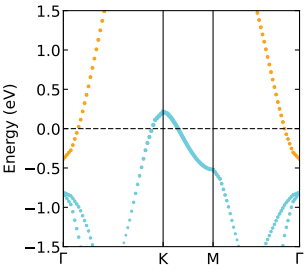

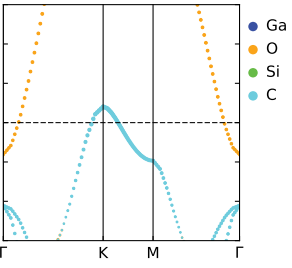

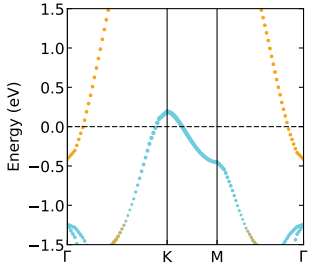

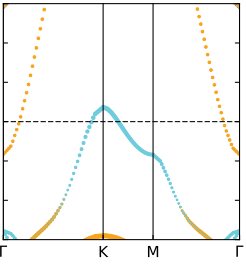


-6% -2%

+2% +6%

**Figure S5.** (Projected) Electronic band diagram of the Ga_2_O_3_/SiC vdW bilayer heterostructure under various biaxial strains applied to it.

**
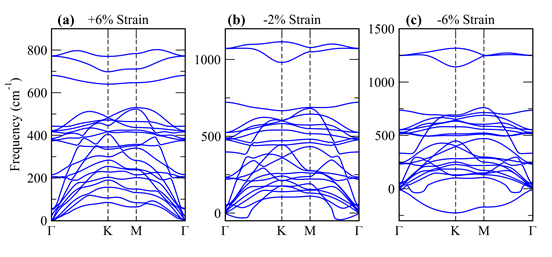
Figure S6:** Phonon dispersion curves of the Ga_2_O_3_/SiC vdW bilayer heterostructure under varying percentage of biaxial strain: (a) +6% Strain, (b) -2% Strain, (c) -6% Strain. ‘+’ corresponds to tensile strain while ‘-’ sign corresponds to compressive strain.

To investigate the dynamic stability of the Ga_2_O_3_/SiC vdW bilayer heterostructure under biaxial strain, we calculated the phonon dispersion relations of the bilayer heterostructure under varying percentages of biaxial strains. Figure S6 depicts the phonon dispersion relations of the Ga_2_O_3_/SiC vdW bilayer heterostructure under strains from −6 to +6%. As the figure suggests, for tensile strain up to +6% biaxial strain, no imaginary frequency exists through the Brillouin zone, indicating the dynamic stability of the bilayer system under tensile strain up to +6%. Nevertheless, while the bilayer system is under compressive biaxial strain (− 2% and -6% biaxial strain), negative frequencies are observed in the Brillouin zone of the phonon spectrum, suggesting that the Ga_2_O_3_/SiC vdW bilayer heterostructure may not be dynamically stable under compressive strain.

**Table S1.** Binding energy, optimized interlayer distance and type of band alignment of the four configurations of the Ga_2_O_3_/SiC vdW bilayer heterostructure.

| Stacking Configuration | Binding energy (meV$/Å$^2^) | Optimized interlayer distance ($Å)$ | Band Alignment |
| --- | --- | --- | --- |
| X_1_ | -28.15 | 3.025 | Type-III |
| X_2_ | -25.05 | 3.183 | Type-III |
| X_3_ | -20.51 | 3.294 | Type-III |
| X_4_ | -20.48 | 3.546 | Type-III |

**References**

1. Liao, Y., Zhang, Z., Gao, Z., Qian, Q. & Hua, M. Tunable Properties of Novel Ga2O3Monolayer for Electronic and Optoelectronic Applications. *ACS Appl Mater Interfaces* **12**, 30659–30669 (2020).

2. Bekaroglu, E., Topsakal, M., Cahangirov, S. & Ciraci, S. First-principles study of defects and adatoms in silicon carbide honeycomb structures. *Phys Rev B Condens Matter Mater Phys* **81**, 1–9 (2010).

3. Şahin, H. *et al.* Monolayer honeycomb structures of group-IV elements and III-V binary compounds: First-principles calculations. *Phys Rev B Condens Matter Mater Phys* **80**, (2009).

4. Ferdous, N., Islam, M. S., Biney, J., Stampfl, C. & Park, J. Two-dimensional SiC/AlN based type-II van der Waals heterobilayer as a promising photocatalyst for overall water disassociation. *Sci Rep* **12**, 1–13 (2022).
